# Correspondence

**Published:** 1890-02

**Authors:** 


### Correspondence.

---

New York, January 14, 1890.

Now that the holiday season is over, medical matters which were held in abeyance for the time being have received a fresh stimulus. The colleges and hospitals are again in full swing and operations and clinics are very numerous. The epidemic of influenza, which is generally known as "la grippe," has been exceedingly prevalent here, attacking all classes and characterized by very great virulence.

This disease seems to have different types of character. Some patients are attacked with a coryzy or a bronchitis while others develop a great soreness of the entire muscular system and complete physical prostration. The range of temperature in this disease varies from 102 degrees to 105 degrees F., but the dangerous element of the disease seems to consist in the pneumonic process which follows in a great many instances and which is, to say the least, very intractable to treatment.

For the bronchitis accompanying this disease, and with which it is in most instances ushered in, Prof. F. Delafield, of the College of Physicians and Surgery, of this city, recommends the following to be taken every hour, day and night, which is to be made into a pill or capsule.

| | | |
|---|---|---|
| ℞. | Extract Belladonnæ, - - - - - - - | gr. 1-40 |
| | Ipicac. pulv., - - : - - - - | gr. 1-20 |
| | Dover's pulv., - - - - - - . - - - | gr. 1-20 |
| | Quinæ sulph., - - - - - - - - - - | gr. 1-4 |

Prof. Gill Wylie, of the New York Polyclinic, calls attention to the fact that a great many females who are treated for uterine diseases, are, in reality, suffering from rectal trouble of some kind, and he states that the bearing down pains which women so fequently complain of, and for the cause of which so many doctors are apt to look to the uterus, are alone due to disease of the rectum. When a patient presents herself at his office for treatment of any trouble relating to the uterus, he has made it a habit to, first of all, insist upon an examination of the rectum. He further says that by treating the rectum alone, he has cured a great many cases of supposed uterine disease, which did not yield to any other form of treatment.

Among the recent operations performed at Roosevelt Hospital, by Dr. McBurney, of the College of Physicians and Surgeons, was one which possesses sufficient scientific interest to narrate in detail: Four months ago a physician, while driving in his buggy, was thrown with some force to the ground, striking upon the side of the head. There were no external marks of violence, but immediately afterwards it was found that he was asphasic and hemiphlegic on the right side. The hemiphlegia, however, gradually improved somewhat, but the asphasia continued to be complete, and showed no signs, whatever, of improvement. To this end it was decided to attempt his relief by trephining the skull at a point directly over the center of speech. The diagnosis in this case was made by Dr. McBurney, assisted by Dr. Starr, of the College of Physicians and Surgeons, of this city.

The wound made in the skull by the trephine being not deemed sufficiently large for all practical purposes, it was further increased by the employment of a Rongeur forceps, and to the great satisfaction of the operator a clot was found dipping down between the sulci of the brain. This was removed by means of a piece of sponge and the wound was then packed with iodoform gauze and a drainage tube was inserted. A week after the operation the patient was able to speak a few words and was steadily improving, the hemiplegia having altogether disappeared.

The operation of suprapubic cystotomy seems to be a favorite one among the surgeons of this city. The old operations, median or lateral lithotomy, are now much less frequently done. The adage, that there is a fashion in medicine as well as in dress, is well illustrated by this one fact. Trendelenberge, who was the first to popularize this operation and to advocate it strongly, in preference to either of the other two methods, recommended that the pelvis of the patient be elevated by placing his legs over the shoulders of an assistant, putting a colpeurynter in the rectum and then filling it with water, so as to push up the bladder. This position is not the one adopted here, the patient merely lying flat upon the operating table. The colpeurynter is, however, used, and from eight to ten ounces of water are thrown into the bladder through a catheter inserted in the urethra. After the incision is made in the median line through the abdominal muscles, the peritoneum is pushed up by means of the finger in the abdominal wound, two sutures are passed through the bladder, and the latter incised between them. The vesical wound is not sutured, but a drainage tube is fastened in the bladder through the incision, which is left to heal by granulation.

Another operation which is being practiced very frequently, is that for the radical cure of hernia. The one which has the preference here is that known as the McBurney method. The essential points in which this differs from the other methods, are that immediate healing of the wound is not only not desired, but is even prevented. The edges of the inguinal ring are not sewn together, but it is desirable that the entire wound should heal by granulation so as to make a very firm cicatrix. To attain this end it is kept packed with iodoform gauze, and required from three to four weeks to heal.

This method of treating hernia has been so successful and free from mortality, that it is commonly resorted to in this city and is equally applicable to the treatment of femoral and inguinal hernia.

Prof. Wyeth, of the New York Polyclinic, in operating upon a patient with cancer of the breast formulated this dictum, that the operator, who in removing the mamma for cancers of that organ, fails to open the axilla and remove any enlarged glands that may exist in that region, is culpable, and deserves censure. Statistics of operations of this kind performed in this manner have shown much better results than in former times.

Dr. Tomson, of the Medical Department of the New York University, recommends for the treatment of asthma, arsenic, in large doses and kept up for at least two years. If tried in this manner, he says it will cure a large proportion of cases. He begins the treatment with two or three drops of Fowler's solution, and increases each dose by a drop and carrying it up till the patient shows the systemic effects of the drug. Then he reduces the amount by several drops, but still keeps on at that dose.

Dr. Weir, of the College of Physicians and Surgeons, of this city, read a paper recently in the Academy of Medicine, bearing on the question whether the administration of ether had any injurious effects on the kidneys or not—though he believes it has been fully settled in the minds of many American surgeons that it does have an injurious effect on the kidneys, he has never, in the experience in surgical cases running over many years, been able to satisfy himself that such was the case. The author quoted a number of instances noted by other observers in which albumen was found in the urine after the administration of ether, but he thinks this was due to the operative procedures that had been carried out rather than to the effect of the anæsthetic. J. P. R.

